# Analysis of Spectral Sensing Using Angle-Time Cyclostationarity

**DOI:** 10.3390/s19194222

**Published:** 2019-09-28

**Authors:** Pedro Souza, Vinicius Souza, Luiz F. Silveira

**Affiliations:** 1 Engineering and Technology Department, Federal Rural University of Semi-Arid, Pau dos Ferros 59900-000, RN, Brazil; 2 Department of Computer Engineering and Automation, Federal University of Rio Grande do Norte, Natal 59064-741, RN, Brazil

**Keywords:** angle-time cyclostationarity, cyclostationarity, cognitive radio, dynamic spectrum access, spectral sensing

## Abstract

This work presents a novel spectral sensing method for the detection of signals presenting nonlinear phase variation over time. The introduced method is based on the angle-time cyclostationarity theory, which applies transformations to the signal to be sensed in order to mitigate the effects of nonlinear phase variation. The architecture is employed for sensing binary phase shift keying (BPSK) signals, being also compared with time cyclostationarity. The obtained simulation results clearly demonstrate the efficiency of the proposed approach, while presenting improved performance in terms of the detection rate of primary users increased by about 8 dB.

## 1. Introduction

The development and widespread use of wireless communication devices has led to development of several studies concerned with the electromagnetic spectrum [1]. Research works demonstrated that the electromagnetic spectrum access relies on more relevant aspects other than its simple limited availability [2]. From this point of view, the dynamic spectrum access (DSA) has been proposed as a novel spectrum allocation policy [3]. In the specific case of radio systems, the transceiver should be able to detect free portions of the electromagnetic spectrum [4], by employing spectrum sensing techniques [5].

In general, three classic methods are defined in the literature for spectrum sensing: detection by energy, analysis of cyclostationary characteristics, and coupled filters [6]. In spectrum sensing by cyclostationary characteristics analysis, detecting the free portions of the spectrum is based on statistical moments of the received signal [7]. This method is regarded as being the most prominent one in scenarios characterized by low signal-to-noise ratios (SNR), because there is no need for the previous knowledge of the signals to be sensed [2].

Recently, some studies analyzed the sensing performance through the use of cyclostationarity in distinct communications scenarios. The authors in [8] proposed the use of a cyclostationary detector based on the softmax regression model, with the objective to improve detection performance under low SNRs in additive white Gaussian noise (AWGN) channels. The work developed in [9] assessed the gains obtained through cooperative sensing of the spectrum in real conditions using mobile sensors randomly displaced in an environment while using cyclostationary analysis. The goal is to achieve better results associated with the degenerative effect on communication channels. The author in [10] investigated the performance of a multiple input, multiple output, orthogonal frequency-division multiplexing (MIMO-OFDM) radio system where the cognitive radio equipment senses the communication channels continuously through compressive sensing with cyclostationary detection. The use of cyclostationary analysis for sensing signals whose sampling rate is lower than the Nyquist rate in AWGN channels is suggested in [11]. Besides, the work in [12] developed a cyclostationary detector for binary offset carrier (BOC) signals, which are widely used in current and next generation global navigation satellite systems. The aforementioned detection technique is also assessed in AWGN channels.

In most of the spectrum sensing architectures based on cyclostationary analysis, the signal to be sensed has a phase angle that varies linearly over time [8,11,12]. However, in communication systems, the received signal has a nonlinear phase variation caused by a time-variant Doppler, thus distorting the cyclostationary features of the analyzed signals [13,14]. Thus, the obtained results become inaccurate, justifying the development of spectrum sensing techniques that can be more effective in this scenario. In the literature, in scenarios where Doppler deviations are considered, the solution to mitigate its effect consists of using multiple receiving antennas [10,15] or in employing cooperative spectrum sensing [9,16,17], which implies higher cost for the system implementation.

This work aims at sensing signals with nonlinear phase behavior with good accuracy using the angle-time cyclostationary (ATCS) analysis. The introduced technique consists of a novel feature extractor to provide a generalized representation of the conventional cyclostationarity concept, which is referred to as time cyclostationarity hereafter.

The use of ATCS processes has been quite successful in the field of mechanical engineering, mainly for extracting signal features in the assessment of variables related to the rotational movement of engines, where speed varies over time [13,18,19,20]. In particular, this work addresses the use of ATCS theory associated with a detection architecture in order to decide whether a communication signal exists or not in a given range of the spectrum. The improved performance of the proposed approach is also validated through a proper comparison with cyclostationarity sensing methods by simulation results. The method does not increase the computational complexity compared to time cyclostationary, being also highly parallelizable analogously to the cyclostationary detection [21,22]. In addition, highly flexibility exists, with the possibility to use it in a cooperative sensing system or with multiple antennas to provide even greater robustness to the effects of Doppler deviations.

The remainder of this work is organized as follows. The theory of angle-time cyclostationary processes, which can be used for spectral sensing, is described in Section 2. Section 3 addresses the proposed technique. Section 4 presents the simulation results, which are discussed in detail. Finally, the main conclusions are given in Section 5.

## 2. Angle-Time Cyclostationary Procedures

Random signal processing generally adopted a model in which the signals are Wide Sense Stationary (WSS) [2]. However, in signals found in wireless communication systems, statistical parameters vary over time. A more effective method for modeling the statistical behavior of these signals is to assume that they are cyclostationary [23]. In this case, some statistical moments can vary over time, but periodically. However, in scenarios characterized by nonlinear phase signals, the cyclostationary characteristics are shaded [18], and the use of angle-time cyclostationary analysis can be more appropriate.

Initially, this section introduces the fundamental concepts about cyclostationary and angle-time cyclostationary analysis. In the following, it is shown how these analysis can be used for spectral sensing. Finally, we present a mathematical demonstration that cyclostationary processes are a special class of angle-time cyclostationary processes.

### 2.1. Cyclostationary Analysis

A given signal x(t) is said to be second-order cyclostationary if its autocorrelation function is periodic in time [24]:

(1) $$R_{x}\left(t+T,\tau \right)=R_{x}\left(t,\tau \right),$$

where *T* is the cyclostationary period, τ is the time delay, and $R_{x}\left(t,\tau \right)$ is the autocorrelation function defined by [25]:

(2) $$R_{x}\left(t,\tau \right)=E\left\{x\left(t\right)x^{*}\left(t-\tau \right)\right\},$$

where $x^{*}\left(t-\tau \right)$ is the conjugate complex of x(t−τ) and E{·} denotes the expected value operator.

When the theory regarding second-order cyclostationary processes is applied to spectral sensing, the main function used is the spectral correlation density (SCD) represented by $S_{x}^{\alpha }\left(f\right)$, which is defined as the double Fourier transform of the autocorrelation function of a cyclostationary process, i.e.,: [26]:

(3) $$S_{x}^{\alpha }\left(f\right)=F_{\begin{array}{c} t\to \alpha \\ \tau \to f \end{array}}\left\{R_{x}\left(t,\tau \right)\right\},$$

where α is the cyclic frequency and *f* is the frequency, both measured in Hz. In this case, the first Fourier transform maps the time for the cyclic frequency α, while the second one maps a time delay τ (in seconds) for the frequency *f*. It is effectively demonstrated in [24] that the SCD can be calculated as the correlation between the spectral components *f* and f+α of a signal x(t), that is:

(4) $$S_{x}^{\alpha }\left(f\right)=\lim_{W\to \infty }\frac{1}{W}E\left[X_{W}^{*}\left(f\right)X_{W}\left(f+\alpha \right)\right],$$

where $X_{W}\left(f\right)$ is the Fourier transform of signal x(t) in a window of finite length *W*.

The evaluation of the SCD as expressed by Equations (3) and (4) generates a surface on the plane (f,α), which is symmetric both in terms of *f* and α [27]. Considering such existing symmetry, a projection of the SCD on a plane orthogonal to *f* can be obtained for α≥0, which is called alpha profile [2]:

(5) $$\rho \;\left(\alpha \right)=\frac{\max_{f}\left[S_{x}^{\alpha }\left(f\right)\right]}{\max_{f,\alpha }\left[S_{x}^{\alpha }\left(f\right)\right]}.$$

### 2.2. Angle-Time Cyclostationary Analysis

Analogously to the case of cyclostationary processes, a given signal x(t) is said to be second-order angle-time cyclostationary if its respective angle-time autocorrelation function is periodic with respect to the angle, that is [13]:

(6) $$R_{x}\left(\theta ,\tau \right)=R_{x}\left(\theta +2\pi ,\tau \right),$$

where $R_{x}\left(\theta ,\tau \right)$ is the angle-time autocorrelation function defined by [19]:

(7) $$R_{x}\left(\theta ,\tau \right)=E\left\{x\left(t\left(\theta \right)\right)x^{*}\left(t\left(\theta \right)-\tau \right)\right\},$$

and t(θ) is a time instant that corresponds to a given angle θ.

When angle-time cyclostationary processes are used, the most important analysis tool is the order-frequency spectral correlation (OFSC) function, which is defined as the double Fourier transform applied to the angle-time autocorrelation function, resulting in [13]:

(8) $$S_{x}^{\alpha_{\theta }}\left(f\right)=F_{\begin{array}{c} \theta \to \alpha_{\theta }\\ \tau \to f \end{array}}\left\{R_{x}\left(\theta ,\tau \right)\right\}.$$

In this case, the first Fourier transform maps the phase θ (in radians) for the cyclic angular frequency $\alpha_{\theta }$ (dimensionless), whereas the second Fourier transform maps a time delay τ (in seconds) for the frequency *f* (in Hz). According to [13] it can be demonstrated that Equation (8) can be rewritten as:

(9) $$S_{x}^{\alpha_{\theta }}\left(f\right)=\lim_{W\to \infty }\frac{1}{\Phi (W)}E\left\{F_{W}{\left[x(t)\right]}^{*}F_{W}\left[x_{\alpha_{\theta }}\left(t\right)\right]\right\},$$

where $F_{W}\left[\cdot \right]$ is the Fourier transform over a finite time window *W* and $x_{\alpha_{\theta }}\left(t\right)$ is a transformed representation of the signal x(t), which is calculated as [19]:

(10) $$x_{\alpha_{\theta }}\left(t\right)=x\left(t\right)e^{-j\alpha_{\theta }\theta \left(t\right)}\dot{\theta }\left(t\right).$$

Besides, the instantaneous angular speed in rad/s, is given by:

(11) $$\dot{\theta }\left(t\right)=\frac{d\theta (t)}{dt}.$$

From $\dot{\theta }\left(t\right)$, it is possible to obtain the angular sector spanned during the time interval *W* in the form:

(12) $$\Phi \left(W\right)=\int_{W}\dot{\theta }\left(t\right)dt.$$

The OFSC definition presented in Equation (9) is similar to the SCD one given in Equation (4). Besides, unlike the SCD, the OFSC corresponds to the statistical correlation between the signal x(t) and its respective transformed version $x_{\alpha_{\theta }}\left(t\right)$, which is calculated from Equation (12).

Analogously to the cyclostationarity and considering the symmetries that exist in the OFSC, the alpha-angle profile can be defined as the projection of the OFSC in a plane orthogonal to *f* for $\alpha_{\theta }\geq 0$, i.e.:

(13) $$\rho_{\theta }\;\left(\alpha_{\theta }\right)=\frac{\max_{f}\left[S_{x}^{\alpha_{\theta }}\left(f\right)\right]}{\max_{f,\alpha }\left[S_{x}^{\alpha_{\theta }}\left(f\right)\right]}.$$

**Figure 1:**
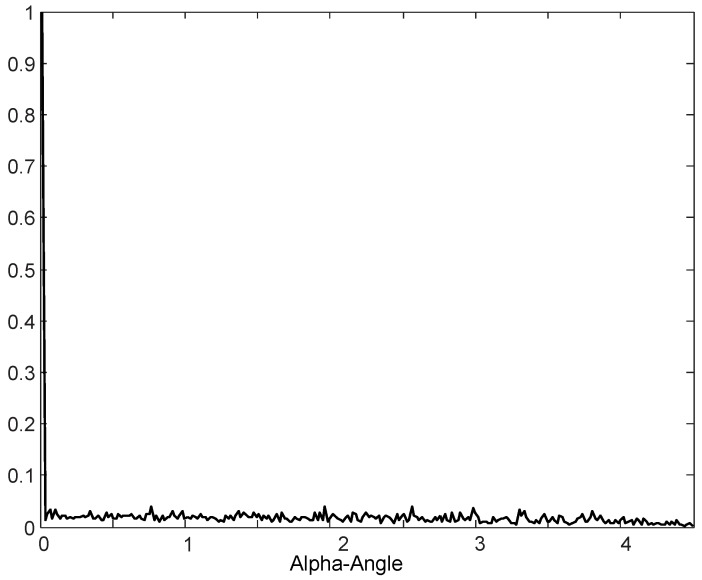
Alpha-Angle profile calculated for a Gaussian noise with zero mean and unit variance.

Spectral sensing based on the Cyclostationary or Angle-Time Cyclostationary analysis relies on the principle that the stationary noise has spectral line only for the cyclic frequency α=0, as shown in Figure 1 [23], which represents the alpha-angle profile calculated for a zero-mean Gaussian noise with unit variance. In the other hand, in the case of modulated signals, the alpha-angle profile always presents spectral lines for at least one value α≠0, which can be used for the sensing task.

### 2.3. Angle-Time Cyclostationary Analysis for Communication Signals

This work proposes the use of angle-time cyclostationary analysis for spectrum sensing. In particular, the OFSC and the alpha-angle profile are adopted to determine the presence or absence of communication signals. Thus, it is demonstrated in this subsection that the OFSC is a generalization of the SCD.

Let us consider a typical passing band communication signal x(t). It is assumed that the phase of this signal represented by θ(t) can be written in terms of a quantity that varies linearly with time, while another one presents a differentiable nonlinear variation, that is:

(14) $$\theta \left(t\right)=\omega_{o}t+℘\left(t\right),$$

where $\omega_{o}$ corresponds to the angular frequency of the carrier signal in rad/s, and ℘(t) is any nonlinear variation with respect to time, caused by the distortions of the communication channel. After some manipulation, the transformed version of the signal x(t) corresponding to $x_{\alpha_{\theta }}\left(t\right)$, as defined in Equation (12), can be calculated as:

(15) $$x_{\alpha_{\theta }}\left(t\right)=\nu \left(t\right)e^{-j\alpha_{\theta }\omega_{o}t},$$

where:

(16) $$\nu \left(t\right)=x\left(t\right)\left[\omega_{0}+\dot{℘}\left(t\right)\right]e^{-j\alpha_{\theta }℘\left(t\right)}.$$

Thus, it is possible to obtain the OFCD as:

(17) $$S_{x}^{\alpha_{\theta }}\left(\omega \right)=\lim_{W\to \infty }\frac{1}{\Phi (W)}E\left\{F_{W}{\left[x(t)\right]}^{*}F_{W}\left[\nu \left(t\right)e^{-j\alpha_{\theta }\omega_{o}t}\right]\right\}.$$

Assuming $F_{W}\left[\nu (t)\right]=V\left(\omega \right)$, applying the frequency shifting property of the Fourier transform to term $F_{W}\left[\nu \left(t\right)e^{-j\alpha_{\theta }\omega_{o}t}\right]$, and substituting $\beta =\alpha_{\theta }\omega_{0}$, it is possible to define the OFSC as:

(18) $$S_{x}^{\beta }\left(\omega \right)=\lim_{W\to \infty }\frac{1}{\Phi (W)}E\left\{X^{*}\left(\omega \right)V\left(\omega +\beta \right)\right\}.$$

Equation (18) corresponds to the calculation of a spectral density cross-correlation function between signals x(t) and ν(t). A practical way to check this similarity is obtained when ℘(t)=0, i.e., when the channel does not cause interference in the phase of the received signal. In this case, $\nu \left(t\right)=\omega_{0}x\left(t\right)$ and $\Phi \left(W\right)=\omega_{0}W$, thus making the OFSC identical to the SCD.

In this sense, the OFSC can be seen as a generalization of the SCD function. For cases where the phase of signal x(t) is linear over time, both metrics are equivalent. However, if the phase of x(t) contains nonlinear components, applying the OFSC will lead to other results than those provided by the SCD, also reinforcing the periodic characteristics of x(t), which are lost when a conventional cyclostationary analysis is employed [13].

**Figure 2:**
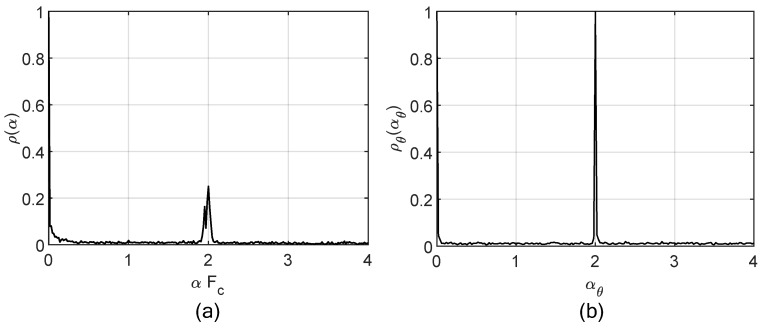
Alpha profile (**a**) and alpha-angle profile (**b**) calculated for a AM-DSB-FC signal with nonlinear phase over time.

To illustrate such concepts, the calculation of the SCD and OFSC are presented in Figure 2a,b, respectively, for an amplitude modulation, double sideband full carrier (AM-DSB-FC) modulated signal with coherent nonlinear phase variation. In this case, the spectral lines of the alpha profile are attenuated, which would impair the detection of this signal during the spectrum sensing. On the other hand, these same spectral rays still exist in the alpha-angle profile, thus denoting the robustness of this metric in scenarios characterized by nonlinear phase over time.

## 3. Proposed Sensing Architecture

The spectrum sensing architecture using the angle-time cyclostationarity analysis proposed this work relies on the detection of amplitude peaks for $\alpha_{\theta }>0$ in modulated signals, since the noise has no amplitude peaks in the alpha-angle profile for $\alpha_{\theta }\neq 0$.

In this context, a detection approach is introduced, based on a decision metric, called sensing metric, calculated from the alpha-angle profile as:

(19) $$\varepsilon =\max\rho_{\theta }\left(\alpha_{\theta }>0\right).$$

Assuming the existence of a communication signal in the aforementioned spectrum range, the value of the sensing metric ε will tend to unit because the alpha-angle profile is normalized in terms of the maximum value of the OFSC. However, if a given spectral band is not occupied, the observed signal will only be composed of white Gaussian noise, thus the value assumed by the sensing metric will tend to lower values as a consequence.

Thus, assuming a suboptimal threshold ξ, the decision on the occupation of a particular spectrum will consist of the following binary hypothesis test:

- If ε<ξ, then the spectral band under analysis is free, and so transmission may occur;
- If ε≥ξ, then the analyzed spectral band is occupied;

In this paper, the suboptimal threshold for decision making ξ is obtained through a curve that relates decision thresholds to the false alarm probabilities of the sensing architecture. This curve is obtained by making the analyzed signal just a AWGN noise with zero mean and unit variance. From the desired False Alarm Probability and using this curve, the threshold to be used is chosen.

### 3.1. Estimation of the OFSC

In this work, the OFSC calculation is carried out through the estimation from a discrete sequence of finite size. It can be demonstrated that the OFSC estimator for a discrete sequence ${\left\{x\left(n\right)\right\}}_{n=0}^{L-1}$ with *L* samples can be determined from the Welch periodogram as [13]:

(20) $${\hat{S}}_{x}^{\alpha_{\theta }}\left(f\right)=\frac{1}{{\Phi S||w||}^{2}}\sum_{s=0}^{S-1}DTFT{\left\{x_{w}\left(n\right)\right\}}^{*}DTFT\left\{x_{\theta w}\left(n\right)\right\},$$

where:

(21) $$x_{w}\left(n\right)=w_{s}\left(n\right)x\left(n\right),$$

(22) $$x_{\theta w}\left(n\right)=w_{s}\left(n\right)x\left(n\right)\dot{\theta }\left(n\right)e^{-j\alpha_{\theta }\theta \left(n\right)},$$

being Φ the angular window given by:

(23) Φ=θ(L−1)−θ(0).

Besides, $w_{s}\left(n\right)$ corresponds to a shifted version in a multiple of *R* samples within a window ${\left\{w\left(n\right)\right\}}_{n=0}^{N_{w}-1}$, i.e., $w_{s}\left(n\right)=w\left(n-sR\right)$, where *S* is given by:

(24) $$S=*\left(\frac{L-N_{w}}{R}+1\right),$$

where $*\left(\cdot \right)$ is the result obtained with the floor operator, DTFT is the discrete-time Fourier transform, and ${\left||w|\right|}^{2}$ is the energy associated with the adopted window.

## 4. Simulation Results

This section presents simulation results to compare the performance of the angle-time cyclostationarity and time cyclostationarity when sensing communication signals. For this purpose, a signal x(t) obtained from BPSK (Binary Phase Shift Keying) modulation subjected to white Gaussian noise is employed, whose phase at the receiver terminal can be determined as:

(25) $$\theta \left(t\right)=2\pi f_{c}t+℘\left(t\right),$$

where $f_{c}$ is the carrier frequency and ℘(t) is the instantaneous phase variation of signal x(t), which is caused by a time-variant Doppler represented by:

(26) $$℘\left(t\right)=A_{d,max}\cos\left(2\pi f_{ad}t\right).$$

In this case, the term $\Delta f_{c}=2\pi f_{ad}A_{d,max}$ corresponds to the maximum Doppler deviation with respect to $f_{c}$ and $f_{ad}$ is the frequency for which the deviation occurs.

The sensing probability of signal x(t) was determined considering a nonlinear variation of the signal phase with $\Delta f_{c}=12$ Hz and $f_{ad}=0.5$ Hz/s. In all cases, the carrier frequency is $f_{c}=4096$ Hz and the sampling frequency is $f_{s}=$32,768 Hz.

The alpha profile was estimated through the cyclic periodogram detection (CPD) algorithm proposed in [28] with the parameters listed in Table 1. The alpha-angle profile was estimated through the Welch periodogram described in Section 3.1 with the parameters presented in Table 2.

### 4.1. Characterization of the Sensing Metric

**Figure 3:**
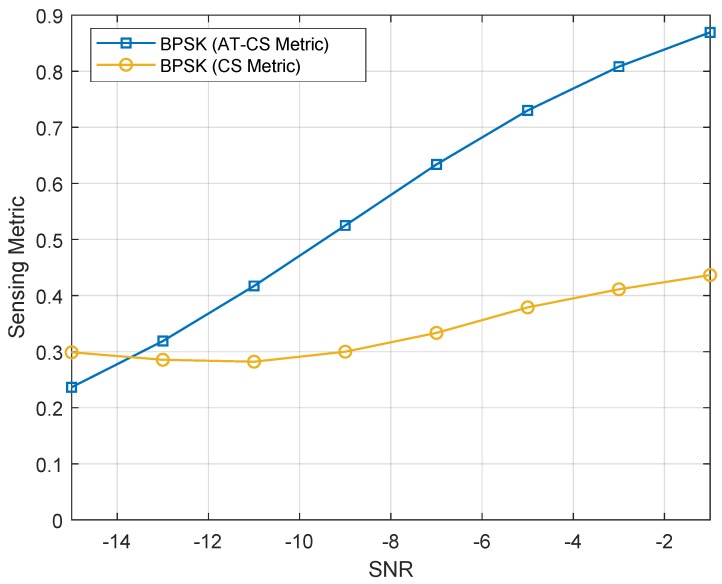
Sensing metrics as a function of the SNR for the conventional cyclostationary and angle-time cyclostationary analysis.

To investigate the behavior of the sensing metric, defined in Equation (19), according to the communication channel, simulation tests were carried out for an SNR range from −15 dB to 0 dB with steps of 1 dB. Besides, for comparison purposes, a reference curve was obtained considering the use of the alpha profile in calculating the sensing metric rather than the alpha-angle profile. The curves were plotted in Figure 3 considering a total of 300 simulations for each value of the SNR, from which the average values were calculated.

From Figure 3, it can be stated that the values of the sensing metrics for both techniques tend to decrease as the noise increases, as it is expected that the performance of the sensing architecture is affected when the SNR decreases. However, it is observed that the value of the sensing metric when using the angle-time cyclostationarity is superior that associated with time cyclostationarity, thus emphasizing the robustness of this sensing technique in scenarios characterized by nonlinear variation of the received signal phase.

### 4.2. Probability of Detection and False Alarm

**Figure 4:**
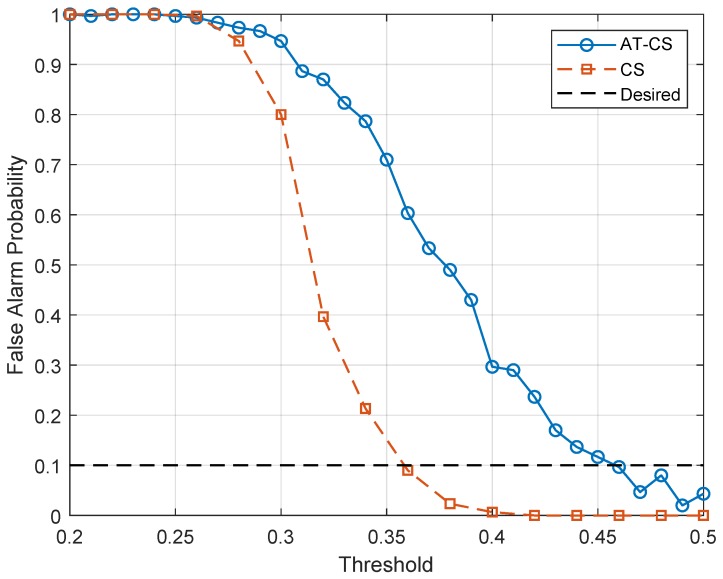
False alarm probability as a function of the detection threshold for the conventional cyclostationary and angle-time cyclostationarity.

Considering standard IEEE 802.22 [29], which discusses the requirements of cognitive radio systems, a constant false alarm probability of 10% is desired. This value can be obtained adjusting the comparison threshold to be used in the decision making of the spectrum sensing architecture. The search for this threshold is performed from the curves plotted in Figure 4, which show the relationship between a given comparison threshold as a function of the false alarm probability for the time cyclostationarity and angle-time cyclostationarity.

**Figure 5:**
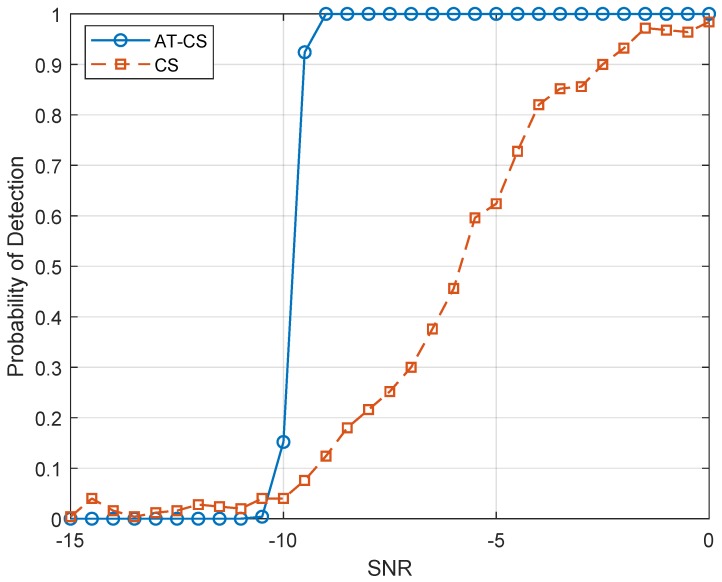
Detection probability of the sensing architecture using the time cyclostationarity and angle-time cyclostationarity.

In Figure 4, the comparison thresholds $\xi_{\mathrm{CS}}=0.358$ and $\xi_{\mathrm{AT}-\mathrm{CS}}=0.464$ were chosen for the time cyclostationarity and angle-time cyclostationarity, respectively, while the performance of the sensing architectures was properly assessed in Figure 5. Each point of the curve in Figure 5 was obtained through 250 simulations, while the average detection rate was determined for each group. It is noted that the angle-time cyclostationarity presents superior performance, being this an expected result due to the behavior of the sensing metrics as a function of the SNR as presented in Figure 3. Once again, this technique proves to be robust when dealing with received signals whose phase variation is nonlinear.

## 5. Conclusions

This paper presented a novel spectrum sensing technique based on the angle-time analysis of communication signals, where this approach is also compared with the conventional time cyclostationary analysis. From the simulation results, it was demonstrated that the angle-time cyclostationarity is highly effective in scenarios where the signal phase does not vary linearly. The main contributions of this work can be stated as follows: (i) application of the angle-time cyclostationarity to spectrum sensing in communication systems; (ii) proposal of a mathematical representation in order to demonstrate that the angle-time cyclostationarity is a generalization of the conventional cyclostationarity; (iii) introduction of a novel sensing metric for the angle-time cyclostationary analysis in terms of the alpha-angle profile; and (iv) development of a novel spectrum sensing technique based on the angle-time cyclostationarity.

Future work includes the extension of this method to sense other types of modulation, such as quadrature phase shift keying (QPSK), quadrature amplitude modulation (QAM), minimum-shift keying (MSK), among others; and development of an automatic modulation classification architecture based on the angle-time cyclostationary analysis.

## Figures and Tables

**Table 1:** Parameters used by the CPD algorithm to estimate the SCD function.

Parameter	Value
Block size (*N*)	512 samples
Number of blocks (*L*)	8 blocks
Smoothing parameter (*M*)	8

**Table 2:** Parameters used by the CPD algorithm to estimate the OFSC function through the Welch periodogram.

Parameter	Value
Block size (*N*)	512 samples
Number of FFT points ($n_{FFT}$)	1024 samples (zero-padding)
Block overlapping	341 samples
Window type	Hanning
$\alpha_{\theta }$ Resolution	0.02

## Abbreviations

The following abbreviations are used in this manuscript:

AM-DSC-FC	Amplitude Modulation, Double Sideband Full Carrier
ATCS	Angle-Time Cyclostationary
AWGN	Additive White Gaussian Noise
BOC	Binary Offset Carrier
BPSK	Binary Phase Shift Keying
DSA	Dynamic Spectrum Acces
MIMO	Multiple Input, Multiple Output
MSK	Minimum-Shift Keying
OFDM	Orthogonal Frequency-Division Multiplexing
OFSC	Order-Frequency Spectral Correlation
QAM	Quadrature Amplitude Modulation
QPSK	Quadrature Phase Shift Keying
SCD	Spectral Correlation Density
SNR	Signal-to-Noise Ratio
WSS	Wide Sense Stationary

## References

[1] Arslan H. (2007). Cognitive Radio, Software Defined Radio, and Adaptive Wireless System. Signals and Communication Technology.

[2] Costa E.L. (1996). Detectional and Identification of Cyclostationary Signals. Master’s Thesis.

[3] Murroni M., Prasad R.V., Marques P., Bochow B., Noguet D., Sun C., Moessner K., Harada H. (2011). IEEE 1900.6: Spectrum sensing interfaces and data structures for dynamic spectrum access and other advanced radio communication systems standard: Technical aspects and future outlook. IEEE Commun. Mag..

[4] Čabrić D., Mishra S., Willkomm D., Brodersen R., Wolisz A. A cognitive radio approach for usage of virtual unlicensed spectrum. Proceedings of the 14th IST Mobile and Wireless Communications Summit.

[5] Mitola J.I. Cognitive Radio. An Integrated Agent Architecture for Software Defined Radio. http://citeseerx.ist.psu.edu/viewdoc/summary?doi=10.1.1.13.1199.

[6] Yucek T., Arslan H. (2009). A survey of spectrum sensing algorithms for cognitive radio applications. IEEE Commun. Surv. Tutor..

[7] Cabric D., Mishra S.M., Brodersen R.W. Implementation issues in spectrum sensing for cognitive radios. Proceedings of the Conference Record of the Thirty-Eighth Asilomar Conference on Signals, Systems and Computers.

[8] Zhang L., Huang H., Jing X. A modified cyclostationary spectrum sensing based on softmax regression model. Proceedings of the 2016 16th International Symposium on Communications and Information Technologies (ISCIT).

[9] Chaudhari S., Kosunen M., Mäkinen S., Oksanen J., Laatta M., Ojaniemi J., Koivunen V., Ryynänen J., Valkama M. (2015). Performance evaluation of cyclostationary-based cooperative sensing using field measurements. IEEE Trans. Veh. Technol..

[10] Rawat D.B. (2016). Evaluating performance of cognitive radio users in mimo-ofdm-based wireless networks. IEEE Wirel. Commun. Lett..

[11] Cohen D., Eldar Y.C. (2017). Sub-Nyquist cyclostationary detection for cognitive radio. IEEE Trans. Signal Process..

[12] Thuillier E., Lundén J. Cyclostationarity-based detection and identification of binary offset carrier-modulated signals. Proceedings of the 2015 23rd European Signal Processing Conference (EUSIPCO).

[13] Abboud D., Baudin S., Antoni J., Rémon D., Eltabach M., Sauvage O. (2016). The spectral analysis of cyclo-non-stationary signals. Mech. Syst. Signal Process..

[14] Proakis J. (1989). Digital Communications.

[15] Li M., Yang D., Lin J., Tang T. Specwatch: Adversarial spectrum usage monitoring in crns with unknown statistics. Proceedings of the IEEE INFOCOM 2016-The 35th Annual IEEE International Conference on Computer Communications.

[16] Gao L., Duan L., Huang J. (2016). Two-sided matching based cooperative spectrum sharing. IEEE Trans. Mob. Comput..

[17] Guan C., Mohaisen A., Sun Z., Su L., Ren K., Yang Y. When smart tv meets crn: Privacy-preserving fine-grained spectrum access. Proceedings of the 2017 IEEE 37th International Conference on Distributed Computing Systems (ICDCS).

[18] D’Elia G., Daher Z., Antoni J. A novel approach for the cyclo-non-stationary analysis of speed varying signals. Proceedings of the ISMA2010 International Conference on Noise and Vibration Engineering.

[19] Baudin S., Rémond D., Antoni J., Sauvage O. (2016). Non-intrusive rattle noise detection in non-stationary conditions by an angle/time cyclostationary approach. J. Sound Vib..

[20] Antoni J. (2007). Cyclic spectral analysis in practice. Mech. Syst. Signal Process..

[21] Lima A.D.L., Silveira L.F.Q., de-Souza S.X. (2018). Spectrum sensing with a parallel algorithm for cyclostationary feature extraction. Comput. Electr. Eng..

[22] Lima A., Barros C., Silveira L., Xavier-de-Souza S., Valderrama C. (2014). Parallel cyclostationarity-exploiting algorithm for energy-efficient spectrum sensing. IEICE Trans. Commun..

[23] Gardner W.A. (1994). Cyclostationarity in Communications and Signal Processing.

[24] Gardner W.A. (1988). Statistical Spectral Analysis: A Nonprobabilistic Theory.

[25] Gardner W.A., Franks L. (1975). Characterization of cyclostationary random signal processes. IEEE Trans. Inf. Theory.

[26] Gardner W.A., Napolitano A., Paura L. (2006). Cyclostationarity: Half a century of research. Signal Process..

[27] Enserink S., Cochran D. A cyclostationary feature detector. Proceedings of the 1994 28th Asilomar Conference on Signals, Systems and Computers.

[28] Zhang Z., Xur X. Implementation of cyclic periodogram detection on VEE for cognitives. Proceedings of the Global Motorsports Congress.

[29] Cordeiro C., Challapali K., Birru D., Shankar S. IEEE 802.22: The first worldwide wireless standard based on cognitive radios. In Proceedings of the First IEEE International Symposium on New Frontiers in Dynamic Spectrum Access Networks.

